# Nocturnal Bee Pollinators Are Attracted to Guarana Flowers by Their Scents

**DOI:** 10.3389/fpls.2018.01072

**Published:** 2018-07-31

**Authors:** Cristiane Krug, Guaraci D. Cordeiro, Irmgard Schäffler, Claudia I. Silva, Reisla Oliveira, Clemens Schlindwein, Stefan Dötterl, Isabel Alves-dos-Santos

**Affiliations:** ^1^Empresa Brazileira de Pesquisa Agropecuária (Embrapa) Amazônia Ocidental, Manaus, Brazil; ^2^Departamento de Ecologia, IBUSP, Universidade de São Paulo, São Paulo, Brazil; ^3^Department of Biosciences, Plant Ecology, University of Salzburg, Salzburg, Austria; ^4^Departamento de Biologia Geral, ICB, Universidade Federal de Minas Gerais, Belo Horizonte, Brazil; ^5^Departamento de Botânica, ICB, Universidade Federal de Minas Gerais, Belo Horizonte, Brazil

**Keywords:** flower signal, pollinator attraction, chemical communication, crop pollination by bees, volatile organic compounds, *Paullinia cupana*, Amazon

## Abstract

Floral scent is an important component of the trait repertoire of flowering plants, which is used to attract and manipulate pollinators. Despite advances during the last decades about the chemicals released by flowers, there is still a large gap in our understanding of chemical communication between flowering plants and their pollinators. We analyzed floral scents of guarana (*Paullinia cupana*, Sapindaceae), an economically important plant of the Amazon, using chemical analytical approaches, and determined the attractiveness of the scent to its nocturnal bee pollinators using behavioral assays in the field. Pollen loads of attracted bees were also analyzed. Inflorescences of guarana emit strong scents, both during day and at night, with some semi-quantitative differences between day- and night-time scents. Synthetic scent mixtures containing some of the identified floral scent components, including the most abundant ones, i.e., linalool and (*E*)-β-ocimene, successfully attracted the nocturnal *Megalopta* bee pollinators. Pollen analyses revealed that many of the attracted bees had pollen grains from previous visits to guarana flowers on their bodies. Overall, our data show that guarana flowers attract nocturnal bee visitors by their strong scents and suggest that the chemical communication between this plant and its pollinators is a key component in crop production of this economically important plant species.

## Introduction

Floral scents are important signals for the attraction of pollinators and may be particularly important for plants pollinated at night when visual signals are of limited use. In the last decades, there has been considerable progress in understanding the chemical communication between nocturnal plants and their pollinators (e.g., moths, beetles, bats; Dobson, 2006; Borges et al., 2016). Just recently, however, a new pollination system mediated by floral scent and involving nocturnal bees as pollinators was described (Cordeiro et al., 2017).

The nocturnal/crepuscular habit has arisen in four families of bees, i.e., Andrenidae, Apidae, Colletidae, and Halictidae, comprising at least 250 species (Warrant, 2007). It is hypothesized that night-active bees evolved this habit as a response to competition, parasitism, and predation during the day (Wcislo et al., 2004). Foraging at night and during crepuscular periods may be beneficial as flowers are often rich in pollen and nectar early in the morning before exploitation by diurnal flower visitors, and late in the evening before night active visitors arrive (“competitor-free space” according to Wcislo et al., 2004; Warrant, 2007). Nocturnal bees must have good vision and a well-developed olfactory system to find their nests and to recognize the flowers at low light intensities (Kelber et al., 2006; Borges et al., 2016). It is likely that they primarily use olfactory floral cues, i.e., floral scents, to efficiently locate appropriate host plants.

**FIGURE 1:**
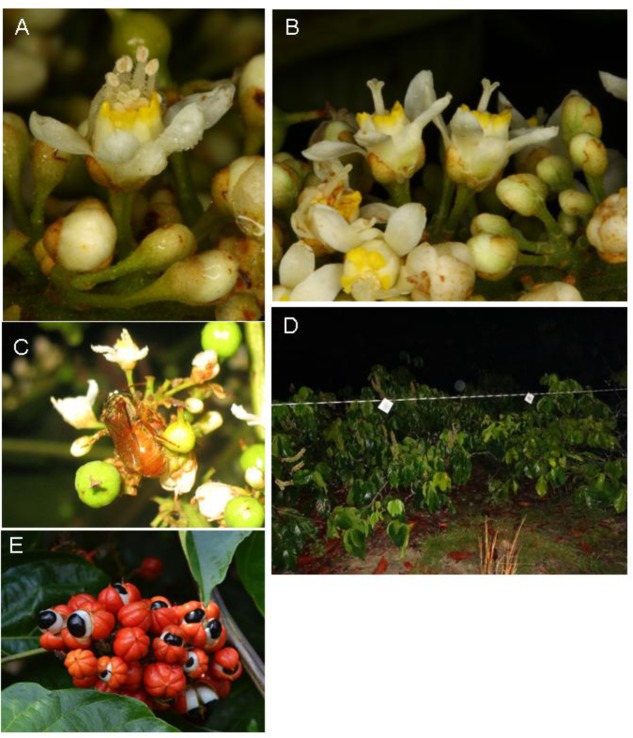
Male **(A)** and female **(B)** guarana flowers. *Megalopta aeneicollis* visiting a guarana flower **(C)**. String and filter papers impregnated with synthetic mixtures or solvent only, as used in the biotests **(D)**. Fruits of guarana **(E)**.

*Paullinia cupana* Kunth (Sapindaceae), popularly named guarana, is an economically important plant of the Brazilian Amazon. The seeds of this plant are used to produce soft drinks, energy drinks, ice creams, creams, pharmaceuticals, and cosmetics (Tavares et al., 2005). Guarana is produced by large and small producers and is one of the most valued products from the Amazon as it is consumed and appreciated by national and international markets. The species is monoecious, but either pistillate or staminate flowers are produced by a given plant individual on a specific day (**Figure 1**). Thus, it depends on cross-pollination to set fruits, with diurnal bees traditionally cited as the main pollinators (Schultz and Valois, 1974; Escobar et al., 1984). Recently, Krug et al. (2015) also reported flower visits by nocturnal and crepuscular bees, which efficiently vector pollen (Krug et al., unpublished data) and are potentially attracted by the floral scent of *P. cupana.*

Here, we collected floral scents of *P. cupana* using dynamic headspace extraction methods and analyzed the samples by gas chromatography/mass spectrometry (GC/MS). We also performed behavioral assays in the field with synthetic compounds and analyzed the pollen load of attracted bees. Specifically, we addressed the following questions: What are the absolute amounts of floral scents released, and does scent differ between day and night in quantitative and compositional (semi-quantitative scent patterns) properties? Are there differences in scent between pistillate and staminate flowers? Are nocturnal/crepuscular bees attracted by the main floral scent components, and if yes, do they carry pollen grains from previous visits to guarana flowers on their bodies?

## Materials and Methods

### Study Species

When cultivated in plantations, *P. cupana* Kunth [=*P. cupana* var. *sorbilis* (Mart.) Ducke] is trimmed to a shrubby habit, despite growing originally as a liana (Castro, 1992). Flowering usually occurs between July and September, which corresponds to the least rainy period in the Amazon region and lasts from five to 45 days. Anthesis begins at night at around 2:00, and the flowers remain open until 10:00–12:00 (Escobar et al., 1984). On any given day, most of the flowers of a guarana plant are functionally either female or male; thus, the sex expression of a single plant changes during its flowering period. Overall, a single plant produces five to six times more staminate than pistillate flowers (Schultz and Valois, 1974). When ripe, the fruits have a reddish peel, a white pulp, and a visible black seed (**Figure 1**; Castro, 1992).

### Study Area

The study was carried out at three guarana plantations in the Amazonas State, Brazil: the Experimental plantation of Embrapa (Brazilian Agricultural Research Corporation) in the city of Manaus (2°53′13.52^″^S/59°58′58.71^″^W), the Experimental field of Embrapa in Maués (3°23′58.20^″^S/57°40′39.95^″^W), and a private commercial plantation (3°21′29.8^″^S/57°42′07.01^″^W) in Maués. At all three sites, the guarana plantations were surrounded by Amazonian Terra-Firme Forest.

### Volatile Sampling and Analyses

Flower volatiles were collected by dynamic headspace extraction methods, as described by Heiduk et al. (2016) in the flowering period of 2014 (October) in Manaus. We bagged inflorescences prior to anthesis on 13 different individuals and collected their scent after the flowers opened. Each inflorescence was sampled twice, at night after flower opening (between 3:30 and 5:30) and during the day (between 7:30 and 9:30), resulting in a total of 26 samples. Seven of the inflorescences had exclusively staminate flowers, and six had exclusively pistillate flowers. To obtain a scent sample, an inflorescence (with 5 to 30 open flowers) was enclosed in a polyester oven bag (10 cm × 20 cm; Toppits^®^) for 2 min. Then, an adsorbent tube was inserted into the bag to collect the volatiles for 2 min using a membrane pump (G12/01 EB; Gardner Denver Thomas GmbH, Fürstenfeldbruck, Germany). The flow was adjusted at 200 ml/min with a flowmeter. The adsorbent tubes (quartz vials, length: 25 mm, inner diameter: 2 mm) were filled with 1.5 mg Tenax^®^-TA (mesh 60–80) and 1.5 mg Carbotrap^®^ B (mesh 20–40, both Supelco). The adsorbents were fixed in the tubes using glass wool. Volatile samples from green leaves were collected with the same method and were used to discriminate between vegetative and flower-specific scent components.

Scent samples were analyzed using GC/MS to (i) identify the flower-specific compounds in the scent samples, and (ii) determine their absolute and relative scent amounts. The system consisted of an automated thermal desorption system (model TD-20, Shimadzu, Japan) coupled to a GC/MS (model QP2010 Ultra EI, Shimadzu, Japan) equipped with a Zebron^TM^ ZB-5 fused silica column (5% phenyl, 95% dimethylpolysiloxane; 60 m long; inner diameter 0.25 mm; film thickness 0.25 μm; Phenomenex), as described in Heiduk et al. (2016). The GC/MS data were processed using the GCMS solution package (Version 4.41, Shimadzu). The identification of compounds was carried out using the Wiley 9, Nist, 2011, FFNSC 2, and Adams (2007) mass spectral libraries, the database available in MassFinder 3, and published data on Kovats retention indices of components. The identity of some of the compounds was confirmed by comparison of mass spectra and retention times with authentic standard compounds.

### Field Bioassays

To test if identified floral volatiles attract the nocturnal/crepuscular bee pollinators, 29 two-choice bioassays were carried out: 12 in Maués in August 2015 (five at the commercial plantation and seven at Embrapa) and 17 in Manaus from September to October of 2015 and 2016. The bioassays in Maués were performed between 3:00 and 6:00, before sunrise (4 × Day-scent 1; 3 × Day-scent 2; see also below), and in the evening between 18:00 and 19:00, after sunset (5 × Day-scent 1; see also below). In Manaus, tests were performed before sunrise between 4:30 and 6:00.

We tested three blends of synthetic scent using compounds identified from guarana flowers. The blends had different concentrations and numbers of compounds, and different relative ratios of compounds. Two of the blends (Day-scent 1, Day-scent 2) resembled, in terms of relative amounts of compounds (as determined by dynamic headspace sampling and GC/MS analysis), the first nocturnal sample analyzed in the lab by GC/MS. After analyzing all diurnal and nocturnal samples, however, this sample was found to be an exceptional night sample as its composition was found to be more similar to the mean diurnal than to the mean nocturnal scent composition of the plant. Thus, we prepared another blend (Night-scent), which resembled the mean nocturnal composition in the relative amounts of the compounds used. Given that, there were no differences in scent between staminate and pistillate flowers (see Results), there was no need to consider the sex of flowers in these blends.

When applied to filter paper, Day-scent 1 released (*E*)-β-ocimene (4%), (*Z*/*E*)-linalool oxide furanoid (5%), methyl benzoate (17%), linalool (64%), epoxy-oxoisophorone (5%), phenylacetonitrile (2%), 4-oxoisophorone (2%), and (*Z*/*E*)-linalool oxide pyranoid (0.4%). Day-scent 2 contained the same component mixture as Day-scent 1 except for (*E*)-β-ocimene and epoxy-oxoisophorone, as these were used up when preparing Day-scent 1 and Night-scent. The composition of Day-scent 2 was as follows: (*Z*/*E*)-linalool oxide furanoid (6%), methyl benzoate (19%), linalool (71%), phenylacetonitrile (2%), 4-oxoisophorone (2%), and (*Z*/*E*)-linalool oxide pyranoid (0.4%). Both Day-scent mixtures were used undiluted for bioassays. The mixture resembling nocturnal scent was used 100-fold diluted in acetone (v/v) and consisted of (*E*)-β-ocimene (57%), (*Z*/*E*)-linalool-oxide (6%), linalool (30%), and (*E*)-β-caryophyllene (7%). The compounds used for the experiments were obtained either from Sigma Aldrich in the highest purity available or were available in the reference collection (built up from various sources; purity >90% each) of the Salzburg lab.

For each choice assay, we offered 50–100 μl (depending on availability) of one of the synthetic mixtures on filter paper (diameter 10 cm; Whatman No. 1), and as negative control either just a filter paper (when using an undiluted mixture) or a filter paper with 50–100 μl of acetone (Sigma-Aldrich, 99.8%) solvent (when using the diluted mixture). As determined by dynamic headspace sampling and GC/MS analysis, the total amount of scent released by our scent baits resembled the scent released by ca. two strongly scented plants at night when using the 100-fold diluted sample, and 200 plants, when using the undiluted samples. The filter papers were tied to a string and hung between two flowering guarana plants (**Figure 1**). The two pieces of filter paper were at least 1 m apart and were also separated by at least 1 m from the guarana plants. When choice assays were performed simultaneously at the same plantation, the distance between assay sites was 10–20 m. Bees hovering within 10 cm of the filter papers or landing on them were collected with an insect net. The collected bees were identified following Santos and Melo (2015). To test whether collected bees visited guarana flowers before responding to the synthetic scents, pollen grains from the bodies of 17 of the bees attracted in Manaus (nine *Megalopta aeneicollis*, one *M. cuprea*, one *M. piraha*, one *M. sodalis*, five *Megalopta* sp.) were sampled and identified. Pollen grains on the bees’ bodies were removed using pincers and fixed in ethanol for 24 h. The material was then centrifuged, the ethanol discarded, and glacial acetic acid (2 ml) was added for another 24 h, before centrifuging again. The pollen grains were then acetolyzed as proposed by Erdtman (1960), which removes the cytoplasmic content, exposing the morphological features, which are useful for pollen identification using a light microscope. The RCPol’s Pollen Collection (online Pollen Catalogs Network^1^) and a specific Amazon Pollen Collection were used for comparison.

### Data Analysis

For analysis of total quantitative (total absolute amount of scent) and semi-quantitative (percentage contribution of single compounds to total scent) differences in scent emission between sexual phases and time periods (night and day), we performed PERMANOVA analyses (fixed factors: *time class, sex*; random factor: *individual* nested in *sex*; 10.000 permutations) based on Euclidean distances and pairwise Bray–Curtis similarities, respectively (Clarke and Gorley, 2006; Primer 6 Version 6.1.15 and Permanova Version 1.0.5). In addition, SIMPER (factor: *time class*) was used in Primer to determine the compounds responsible for semi-quantitative differences in scent between day and night. A PERMDISP in Primer tested whether dispersion differed between day and night scents.

The field bioassay results were analyzed by exact binomial tests of goodness-of-fit using the spreadsheet provided by http://www.biostathandbook.com/exactgof.html (accessed 20 May 2017). Responses toward specific scent blends (pooled number of attracted bees over replicate assays per blend) were compared to responses toward the negative control. Due to the small number of bees attracted to Day-scent 1 (see Results), no statistical test was performed to compare the attractiveness of this mixture to the control.

## Results

### Floral Volatiles

The inflorescences emitted an amount of roughly 200 ng of scent per flower per hour (**Table 1**). Overall, we did not find differences in total absolute amounts of scent per flower between the sexes (Pseudo-*F*_1,25_ = 0.46, *p* = 0.72) or between day and night samples (Pseudo-*F*_1,25_ = 3.28, *p* = 0.10), despite some variation in mean and maximum values (**Table 1**). Similarly, there was a non-significant interaction effect of these factors (Pseudo-*F*_1,25_ = 1.15, *p* = 0.33).

**Table 1:** Mean (minimum, maximum) total absolute and relative amount of each compound detected in the night and day floral scent samples of *Paullinia cupana* var. *sorbilis* (*N* = 13 individuals).

Total absolute amount of scent (ng per hour and flower)	KRI	Night Mean (min/max) 60.32 (3.36/454.53)	Day Mean (min/max) 360.1 (8.74/2376.35)
*Aromatics*			
2-Phenylethanol^∗^	1119	0.00 (0.00/0.03)	0.13 (0.00/0.65)
*Monoterpenes*			
(*E*)-β-Ocimene^∗^	1050	**46.08** (0.04/94.91)	**19.68** (6.06/31.89)
(*Z*)-Arbusculone	1056	0.11 (0.00/0.64)	0.78 (0.00/3.60)
(*E*)-Arbusculone	1074	0.01 (0.00/0.07)	0.11 (0.00/0.50)
(*Z*)-Linalool oxide furanoid^∗^	1078	3.07 (0.00/38.92)	0.55 (0.00/0.85)
(*E*)-Linalool oxide furanoid^∗^	1093	1.95 (0.00/23.07)	2.42 (0.00/6.05)
Linalool^∗^	1100	**24.16** (2.31/50.10)	**60.97** (38.82/82.43)
1,3,8-*p*-Menthatriene	1125	2.93 (0.00/11.96)	1.14 (0.00/4.58)
*Allo*-Ocimene^∗^	1131	0.06 (0.00/0.66)	0.18 (0.00/0.41)
Lilac aldehyde A^∗^	1148	0.03 (0.00/0.34)	–
Lilac aldehyde B+C^∗^	1157	0.03 (0.00/0.33)	0.05 (0.00/0.31)
Lilac aldehyde D^∗^	1172	0.00 (0.00/0.02)	0.00 (0.00/0.03)
(*Z*)-Linalool oxide pyranoid^∗^	1177	–	0.01 (0.00/0.04)
(*E*)-Linalool oxide pyranoid^∗^	1180	0.01 (0.00/0.17)	0.08 (0.00/0.39)
Lilac alcohol A^∗^	1208	0.02 (0.00/0.17)	0.18 (0.00/1.06)
Lilac alcohol B+C^∗^	1218	0.63 (0.01/3.79)	1.74 (0.01/3.79)
Lilac alcohol D^∗^	1232	0.04 (0.00/0.20)	0.02 (0.00/0.19)
*N-bearing compounds*			
Phenylacetonitrile^∗^	1144	0.25 (0.00/1.10)	0.79 (0.00/2.72)
Indole^∗^	1288	0.00 (0.00/0.02)	0.01 (0.00/0.06)
*Irregular terpenes*			
(*E*)-4,8-Dimethyl-1,3,7-nonatriene^∗^	1117	0.13 (0.00/1.06)	0.38 (0.00/1.62)
4-Oxoisophorone^∗^	1149	0.04 (0.00/0.37)	**2.79** (0.00/20.43)
Epoxy-oxoisophorone^∗^	1137	**5.40** (0.00/30.62)	**4.39** (0.00/8.44)
*Sesquiterpenes*			
α-Cubebene	1364	4.27 (0.00/42.46)	0.47 (0.00/4.23)
α-Copaene^∗^	1394	2.39 (0.00/23.32)	0.49 (0.00/3.17)
(*E*)-β-Caryophyllene^∗^	1444	**5.28** (0.00/38.56)	1.98 (0.00/15.18)
α-Bergamotene^∗^	1436	0.00 (0.00/0.02)	0.00 (0.00/0.05)
α-Humulene^∗^	1478	0.23 (0.00/1.71)	0.01 (0.00/0.17)
β-Copaene	1485	0.05 (0.00/0.23)	0.01 (0.00/0.12)
Germacrene D^∗^	1504	0.19 (0.00/1.51)	0.04 (0.00/0.61)
(*E*,*E*)-α-Farnesene^∗^	1504	0.07 (0.00/0.94)	0.01 (0.00/0.21)
*Miscellaneous*			
2,2,6-Trimethyl-3-keto-6-vinyltetrahydropyran	1113	0.02 (0.00/0.25)	0.01 (0.00/0.04)
*Unknown compounds*			
10 Unknown compounds		0.76 (0.00/4.96)	0.16 (0.00/1.00)


*^∗^Identity of compounds was confirmed with synthetic standards compounds. Compounds are listed according to chemical class. KRI, Kovats Retention Index. The four most abundant compounds each in night and day samples are in bold.*

In total 41 compounds of six chemical classes and unknowns were found in the night and day samples. The volatiles consisted of monoterpenes (16 compounds), sesquiterpenes (8), unknown compounds (10), irregular-terpenes (3), N-bearing compounds (2), aromatics (1), and miscellaneous (1) compounds (**Table 1**). Most of the compounds were found both in day and night samples, with only one minor compound each being time class specific (**Table 1**).

There were no differences in the percentage contribution of single compounds between the sexes (Pseudo-*F*_1,25_ = 1.27, *p* = 0.25), but differences were evident between samples collected during the day or the night (Pseudo-*F*_1,25_ = 9.26, *p* = 0.001) with a non-significant interaction (sex × time) effect (Pseudo-*F*_1,25_ = 0.61, *p* = 0.65). A PERMDISP analysis revealed that dispersion differed between day and night scents (*F*_1,24_ = 30.01, *p* < 0.001) with night scents being more variable than day scents (see also **Table 1**). Based on a SIMPER analysis, linalool and (*E*)-β-ocimene were responsible for more than 60% of the observed differences among night and day samples with linalool being more abundant during day-time, and (*E*)-β-ocimene being more abundant at night (**Table 1**).

### Field Bioassays

Thirty-three bees, all *Megalopta*, from at least four species responded in the field bioassays: *M. aeneicollis* (25 individuals), *M. cuprea* (1), *M. piraha* (1), and *M. sodalis* (1). Five of the attracted individuals could not be assigned to a species. The bees flew directly to or around the filter papers with synthetic scent and sometimes even landed on them, whereas no bee approached the negative controls. Three bees responded to Day-scent 1 (an un-diluted mixture). Twelve bees were attracted by Day-scent 2 (an un-diluted mixture) and 18 bees by the night-scent (a 100-fold diluted mixture) with these two scent mixtures being significantly more attractive than the controls (*p* ≤ 0.001 for both; **Table 2**).

**Table 2:** Number of *Megalopta* individuals attracted to different scent mixtures in field bioassays performed in Manaus and Maués.

Bees	Day-scent 1	Day-scent 2^∗^		Night-scent^∗^

	Maués *N* = 9	Manaus *N* = 9	Maués *N* = 3	Manaus *N* = 8
*Megalopta aeineicollis*		9		16
*Megalopta cuprea*		1		
*Megalopta piraha*				1
*Megalopta sodalis*				1
*Megalopta* sp.	3		2	


*No bees responded to negative control treatments. *N* refers to the number of trials. ^∗^Scent treatment attracted significantly more bees than the control (exact binomial test, *p* < 0.01).*

### Pollen Analyses

From the 17 *Megalopta* bees whose pollen loads were analyzed, six (three *M. aeneicollis*, one *M. piraha*, two *M*. sp.) carried only pollen grains of *P. cupana* on their body. Four bees carried pollen of *P. cupana* as well as pollen of other species (two carried pollen of Arecaceae and two of *Croton*). No pollen was found on the seven other bees collected.

## Discussion

Our study shows that inflorescences of guarana emit strong scents, which do not differ in quantitative and semi-quantitative properties between the sexual phases, but do differ in semi-quantitative properties between day- and night-time. Despite linalool and (*E*)-β-ocimene being the two most abundant compounds in both time periods, the mean value for (*E*)-β-ocimene was higher at night than during the day, and the mean value for linalool was higher during the day than at night. Both synthetic day and night scents attracted nocturnal *Megalopta* bees of different species.

Linalool and (*E*)-β-ocimene are two of the most widespread compounds among floral scents (Knudsen et al., 2006), and this is also true for plants pollinated by nocturnal visitors, such as moths or bats (Dobson, 2006). In several such plants, these two compounds are, similar to guarana, the most abundant compounds (Dobson, 2006). Interestingly, however, the scent of guarana strongly differs from that of the Myrtaceae species *Campomanesia phaea*, which is primarily pollinated by nocturnal bees, such as *Megalopta* and *Ptiloglossa* species. *C. phaea* mainly releases aliphatic and aromatic compounds (e.g., 1-octanol, 2-phenylethanol; Cordeiro et al., 2017) showing that flowers attracting nocturnal bees may have quite different scents.

Our data show that pistillate and staminate flowers release the same scents. Because the bees (females and males) indiscriminately visit pistillate and staminate flowers of guarana exclusively to gather nectar (Krug, unpublished data), this seems to be a good strategy for the plant in order to equally attract the pollinators to flowers of both sexes (Tollsten and Knudsen, 1992). In the bioassays, 33 individuals of nocturnal bees were attracted to the synthetic floral scent mixtures, all *Megalopta*. These bees are the first visitors to arrive at guarana flowers after they open, and efficiently vector pollen (Krug et al., unpublished data). Curiously, *Ptiloglossa lucernarum*, another nocturnal/crepuscular bee which commonly visits guarana flowers (Krug et al., 2015) and was observed visiting flowers during our bioassays, was not attracted to the bait. This suggests that our synthetic mixtures were lacking components needed to attract *Ptiloglossa* bees or that visual cues were missing. This result contrasts with findings of Cordeiro et al. (2017), in which *Ptiloglossa* but no *Megalopta* bees were attracted to synthetic scents of *C. phaea*.

Of the four *Megalopta* species identified among the attracted individuals, two (*M. aeineicollis, M. sodalis*) are known visitors of guarana flowers (Krug et al., 2015). Moreover, our pollen analyses show that several of the attracted bee individuals in our bioassays had visited flowers of guarana before, suggesting that the compounds used in the experiments are key signals for attracting *Megalopta* bees to guarana flowers. The bees responded to different compositions and concentrations of compounds, pointing toward an olfactory circuit that is not highly specialized. Indeed, *Megalopta* bees are known to be generalists and visit a large number of plant species from various families as demonstrated by analyses of pollen grains from brood cells in nests of two *Megalopta* species in Panama (Wcislo et al., 2004). Along these lines, some of the bees collected in present study carried pollen from multiple families. We speculate that, like pollen-collecting diurnal bees (Dobson, 2006), nocturnal bees function as pollen dispersers of a diverse group of plants. However, we urgently need more data on nocturnal bee and plant interactions, as well as the mechanisms of communication between nocturnal bees and their host plants to better understand the biology and ecology of this group of insects. Future studies should also determine the relative contribution of diurnal and nocturnal bee visitors to fruit set in guarana, and the importance of the diurnal floral scents of this plant for attracting the diurnal visitors. Given that flowers release high amounts of linalool, a compound known to be an attractant for various diurnal bees (e.g., Dötterl and Vereecken, 2010) during the day, it is very likely that floral scents also play a key role in the attraction of diurnal pollinators to guarana flowers. Thus, available data suggest that the chemical communication between this plant and its pollinators is a key component in crop production of this economically important plant species.

## Author Contributions

CK, GC, RO, CS, SD, and IAS collected the data and made the biotests in the field. GC, IS, and SD analyzed the chemical data. CIS analyzed the pollen samples. CK, GC, SD, and IAS wrote the manuscript. All the authors improved the manuscript with comments.

## Conflict of Interest Statement

The authors declare that the research was conducted in the absence of any commercial or financial relationships that could be construed as a potential conflict of interest.

## Footnotes

1 www.rcpol.org.br
